# Selective Brain Hypothermia in Acute Ischemic Stroke: Reperfusion Without Reperfusion Injury

**DOI:** 10.3389/fneur.2020.594289

**Published:** 2020-11-13

**Authors:** Jae H. Choi, Sven Poli, Michael Chen, Thanh N. Nguyen, Jeffrey L. Saver, Charles Matouk, John Pile-Spellman

**Affiliations:** ^1^Neurovascular Center, Neurological Surgery, P.C., Lake Success, NY, United States; ^2^Hybernia Medical, LLC, New Rochelle, NY, United States; ^3^Department of Neurology & Stroke, Hertie-Institute for Clinical Brain Research, Eberhard-Karls University of Tübingen, Tübingen, Germany; ^4^Stroke Center, Department of Neurosurgery, Rush University Medical Center, Chicago, IL, United States; ^5^Interventional Neurology/Neuroradiology, Boston University School of Medicine, Boston, MA, United States; ^6^Comprehensive Stroke Center and Department of Neurology, University of California, Los Angeles (UCLA), Los Angeles, CA, United States; ^7^Neurovascular Surgery, Department of Neurosurgery, Yale University-New Haven Hospital, New Haven, CT, United States

**Keywords:** stroke, reperfusion, reperfusion injury, hypothermia, brain cooling, selective endovascular brain cooling, neuroprotection

## Abstract

In acute ischemic stroke, early recanalization of the occluded artery is crucial for best outcome to be achieved. Recanalization aims at restoring blood flow to the ischemic tissue (reperfusion) and is achieved with pharmacological thrombolytic drugs, endovascular thrombectomy (EVT) devices, or both. The introduction of modern endovascular devices has led to tremendous anatomical and clinical success with rates of substantial reperfusion exceeding 80% and proven clinical benefit in patients with anterior circulation large vessel occlusions (LVOs). However, not every successful reperfusion procedure leads to the desired clinical outcome. In fact, the rate of non-disabled outcome at 3 months with current EVT treatment is ~1 out of 4. A constraint upon better outcomes is that reperfusion, though resolving ischemic stress, may not restore the anatomic structures and metabolic functions of ischemic tissue to their baseline states. In fact, ischemia triggers a complex cascade of destructive mechanisms that can sometimes be exacerbated rather than alleviated by reperfusion therapy. Such reperfusion injury may cause infarct progression, intracranial hemorrhage, and unfavorable outcome. Therapeutic hypothermia has been shown to have a favorable impact on the molecular elaboration of ischemic injury, but systemic hypothermia is limited by slow speed of attaining target temperatures and clinical complications. A novel approach is endovascular delivery of hypothermia to cool the affected brain tissue selectively and rapidly with tight local temperature control, features not available with systemic hypothermia devices. In this perspective article, we discuss the possible benefits of adjunctive selective endovascular brain hypothermia during interventional stroke treatment.

## Introduction

Several randomized controlled clinical trials have demonstrated the clinical benefit of endovascular thrombectomy (EVT) in selected patients with acute ischemic stroke due to large vessel occlusions (LVO-AIS) (1–7). The introduction of endovascular clot extraction has not only led to tremendous improvements in reperfusion rates (often exceeding 80%) and clinical outcome (compared to intravenous rtPA alone), but also offered opportunities to further refine patient selection criteria and extend the treatment time window (8–10). Although a significant improvement in clinical outcome after LVO-AIS could be achieved, a wide gap remains between the success in restoring perfusion through the occluded artery (>4 out of 5) and the rate of excellent, non-disabled (modified Rankin Scale score 0-1) functional outcome achieved in only ~1 out of 4 treated patients (11). Recently, Van Horn and colleagues evaluated 123 consecutive patients at a single German center from 2015 to 2019 who had complete TICI (Thrombolysis in Cerebral Infarction score) 3 reperfusion and still found 54.5% to have poor clinical outcomes at 90 days (12).

Brain ischemia triggers a cascade of molecular and cellular mechanisms many of which have been identified (13). Following the quick depletion of oxygen and energy carriers from brain tissue it comes to progressive failure of cellular ion pumps, NMDA (N-Methyl- d-aspartate) receptor activation, and anoxic depolarization that further lead to disturbance of ion homeostasis, excitotoxicity, acidification, and increasing cellular influx of Ca2+ (14–17). Activation of nitric oxide synthase and cyclooxygenase-2, generation of free radicals, upregulation of cell adhesion molecules, and increase in the production of proinflammatory cytokines follow (18–22). The resulting inflammatory reactions include recruitment of cell-mediated immunity, activation of protein kinases and matrix zinc-metalloproteinases, and neutrophil transmigration, among others (22–28). In addition, apoptosis is promoted by up-regulation of the BAX (Bcl-2 Associated X-protein) and calpain genes (29, 30).

As a result of these molecular pathways, functional and structural changes follow, such as impaired vasomotor regulation (31, 32), cytotoxic and vasogenic edema (33, 34), and breakdown of the blood-brain-barrier (35–37). With sustained activation of these pathways the risk for extensive neuronal cell death, infarct progression, and intracranial hemorrhage increases (38, 39).

Paradoxically, reperfusion of the ischemic brain tissue can exacerbate these destructive processes that have been triggered by stroke. This is called reperfusion injury and is thought to be the result of multiple pathways of tissue insult, oxidative stress, leukocyte infiltration, complement activation, mitochondrial dysfunction, platelet activation and aggregation, and blood-brain-barrier disruption, culminating in neuron death, brain edema or hemorrhagic transformation (13, 40–42). Reperfusion injury is a common biologic phenomenon across multiple organs and not limited to reperfusion procedures of the neurovasculature, also occurring following treatment of ischemic conditions of the limbs, gastrointestinal tract, and the heart (43–45). The most feared consequence of cerebral reperfusion injury is intracerebral hemorrhage (ICH) (46, 47).

EVT devices are well-suited to remove the target thrombus and anatomically clear the artery to restore blood flow, but do not offer direct therapy of metabolic consequences of ischema. For ameliorating metabolic disruptions, therapeutic hypothermia has been one of the most promising concepts based on its pleiotropic mechanisms of action (48, 49). In this perspective article we present the possible benefits of a novel form of therapeutic hypothermia: endovascular selective brain cooling, and how its adjunct application during endovascular stroke treatment could improve the outcome in LVO-AIS patients by reducing the deleterious impact of ischemia and reperfusion injury.

## Subsections

### The Physiological Limits of Endovascular Clot Extraction

Although there are numerous endovascular devices available to remove a clot from the neurovasculature, in principle, they are of two main types (1–7). One has a tip with a stent-like mesh that lodges into the clot and allows its retrieval and the other, is an aspiration catheter that applies a suction force to the clot while it is removed from the vasculature. The success rate to clear the artery from a clot with endovascular devices is high (Thrombolysis in Cerebral Infarction Scale score 2b and 3) and ranges between 59 to 88% (1–7).

The clinical benefit of the combined treatment approach, i.e., systemic pharmacological therapy and endovascular clot extraction, over pharmacological therapy alone, is due to its high effectiveness to anatomically revascularize the occluded artery. In addition, endovascular catheters may be used to locally infuse fluids and various drugs. However, drugs that are considered neuroprotective and for intra-arterial use are rather limited (50), and currently, there are no intra-arterial agents with the FDA-approved indication to be used for the treatment of acute ischemic stroke.

The rate for symptomatic ICH, often seen with parenchymal hemorrhage, following the combined treatment of acute ischemic stroke varies and may be as high as 10% (1–10). The rate for asymptomatic ICH, often associated with hemorrhagic infarction-type hemorrhagic transformation, is generally higher and may involve as many as 1 out of 3 treated patients (36–39). Clinical factors, such as thrombolytic therapy, thromboembolism, and specific imaging markers, comorbid factors, and clinical work-flow performance markers are often considered risk factors for post-treatment ICH (13, 36–39, 51). As such, these factors represent parts of the puzzle that complete the picture to understanding how stroke evolves toward a critical level of impairment of cerebral autoregulation, edema, blood-brain-barrier disruption, and post-treatment reperfusion injury. Recanalization therapy, albeit necessary and often successful to restore the ischemic brain to baseline condition, does not directly modify these pathophysiologic mechanisms of stroke and reperfusion injury. Furthermore, almost all drugs with mechanisms thought to counter a specific part of the stroke injury cascade have failed to provide a conclusive neuroprotective effect in clinical studies (50, 52). Certainly, a treatment concept that can attenuate or prevent these pathophysiologic processes is desirable and would be an ideal candidate for adjunctive application.

### The Physiological Limits of Systemic Hypothermia

Therapeutic hypothermia has been identified as one of the most promising neuroprotective methods. Systemic cooling experiments in stroke models involving animals across various species have shown that hypothermia is neuroprotective in terms of reduction in infarct size and improvement in neuro-behavioral testing scores (average effect size 44% [95% confidence interval, 40−47%]) without increasing the risk for ICH (48). The clinical translation of systemic hypothermia (mild to moderate hypothermia, reduction of body core temperature to ~33°C −35°C) has been successful in comatose patients with out-of-hospital cardiac arrest in both shockable and non-shockable rhythms (53, 54) as well as in neonates with ischemic encephalopathy (55). In addition, cardiac surgery has been routinely performed with extracorporeal blood cooling or total exchange with cold fluids (profound hypothermia, reduction of body core temperature to ≤25°C) to protect the brain from ischemic injury during circulatory arrest or vascular clamping (56).

The neuroprotective effect of cooling is the result of hypothermia's pleiotropic mechanisms of action. Among a plethora of demonstrated effects that help to produce a physiological state of ischemic tolerance, cooling causes metabolic depression reducing the cellular demand for oxygen and energy, has anti-excitotoxic, anti-inflammatory, anti-edematous, and anti-apoptotic properties, and suppresses the breakdown of the blood-brain-barrier (49, 57, 58). Perhaps the most known endogenous feature of hypothermia is found in hibernating mammals that are able to survive prolonged periods of hypometabolism and reduced tissue perfusion under extreme hypothermic conditions (body temperature decreases even below the freezing point of water) (59).

**Figure 1:**
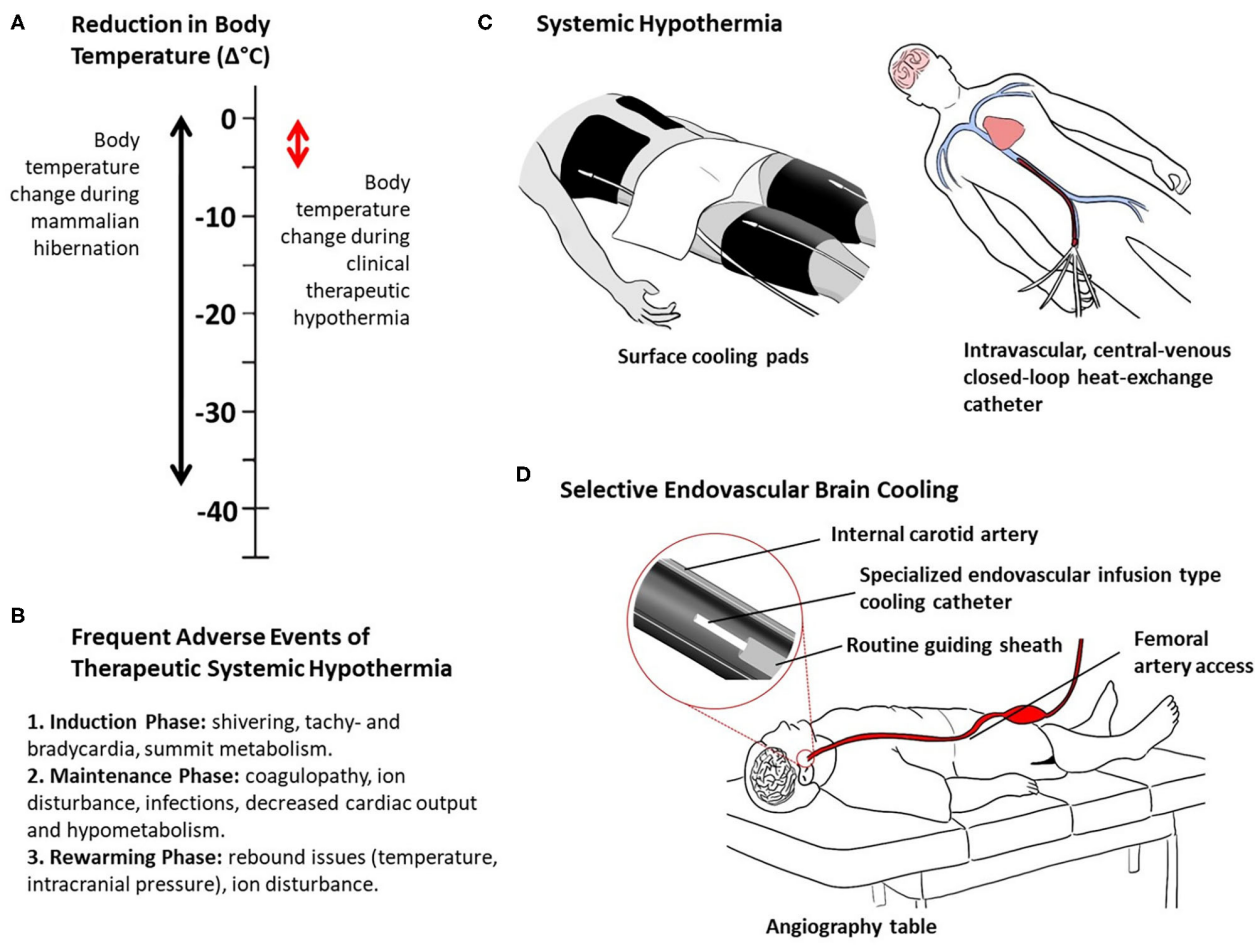
Thermal characteristics of mammalian hibernation and clinical hypothermia **(A)**, frequent adverse events of systemic therapeutic hypothermia **(B)**, and methods of clinical therapeutic hypothermia **(C, D)**. Systemic hypothermia, examples of surface and intravascular conductive cooling **(C)**. Selective endovascular brain cooling, example of direct intra-arterial cold infusion **(D)**.

What appears to be simply natural and is repeated year after year in hibernating mammals, in humans the process of inducing and maintaining systemic hypothermia are extremely difficult, complicated, prolonged, and painful due to the strong physiological counter mechanisms and frequent adverse events (60, 61) (Figures 1A–C). As such, this clinical process requires specialized intensive care resources, sedation, and muscle relaxation, machine ventilation, and co-treatment of the frequent adverse effects of body hypothermia. Despite the successes achieved in the clinical settings of out-of-hospital cardiac arrest and neonatal asphyxia, systemic hypothermia has been difficult to realize in acute stroke patients (62).

### The Promises of Adjunct Endovascular Brain Hypothermia

Selective brain hypothermia is an attractive alternative to systemic hypothermia, providing focused cooling of the injured organ and avoiding the complications of systemic hypothermia, including intubation, shivering, pneumonia, altered coagulability, and cold-induced stress reactions (58). A variety of devices and strategies have been developed to induce selective brain hypothermia and tested in preclinical models and early human clinical trials, including intranasal selective hypothermia, transvenous endovascular cooling, extraluminal vascular cooling, and epidural cerebral cooling (63–72). However, advance of these devices in clinical development has been constrained by slow onset of cooling induced by external cerebral cooling techniques and procedural time delay needed to place internal nasopharyngeal cooling devices. A promising emerging approach that overcomes these limitations is selective endovascular brain cooling (SEBC; Supplement Table) through an interventional catheter system that is navigated to the common or internal carotid artery to perform an intravascular heat-exchange with the arterial blood before it enters the cerebral vasculature (Figure 1D). Different endovascular local heat-exchange concepts exist exploiting the physical forces of conduction or direct mixing of cold fluids with blood, and intravascular vs. extracorporeal heat-exchange methods (63, 72).

Experiments in animal stroke models, commonly with transient occlusion of the middle cerebral artery, have found a significant improvement in outcome (stroke volume, edema, behavioral scores) with brief endovascular selective brain cooling [average effect size 51% (95% CI, 38–64%)] (73, 74). Hereby, the risk for ICH was not increased in the cooling groups compared to control groups. The preferred method is direct intra-arterial infusion of cold fluids to cool the blood that enters the brain either during ischemia, post reperfusion, or during both phases. Although brain cooling was performed for only a short duration, the rapid induction of brain hypothermia led to the suppression of the pathophysiological mechanisms of ischemia and reperfusion injury to levels that allowed a significantly better restoration of the affected brain tissue when compared to the normothermia group.

Speed of brain cooling is one of the unique features of SEBC that distinguishes itself from any form of systemic hypothermia. This is because only a selected vascular territory of the brain is cooled, rather than the whole body, and a direct modification of the arterial input temperature is performed (63). The cooling performance with SEBC is in the range of minutes with the direct infusion method, not hours (Figure 1D). For instance, a brain temperature reduction of more than 3.5°C in 10 min was shown in a large pig model with SEBC using the direct intra-arterial cold infusion (IACI) method (75). In a canine transient occlusion model, a reduction in brain temperature of >10°C within 10 min was found using SEBC, with direct IACI during the phases of ischemia and post-reperfusion (76). The animals with brain cooling had smaller infarct sizes compared to controls. Extracorporeal blood cooling is a more invasive cooling concept primarily performed in the clinical setting of cardiac surgery. Blood from the femoral artery, aorta, or carotid artery is passed through an extracorporeal heat-exchanger where it is cooled to the desired temperature. This cooled and medically processed blood is infused back into the cerebral circulation, often via the carotid artery. In another swine stroke model experiment, extracorporeal blood cooling led to a brain temperature decrease of >6°C in ~30 min (77). In contrast to SEBC with IACI (cold fluid or cold blood), local blood cooling via conduction, i.e., with a closed-loop intra-carotid catheter in which cold fluids circulate, is far less effective due to the limited dimensions of the parent artery and resulting limitations to the size and surface area of the heat-exchange catheter (78).

SEBC has also been studied in humans (Table 1). The feasibility and safety of SEBC with a brief intra-carotid infusion of cold fluid was demonstrated in a first-in-human study in 18 elective cerebrovascular patients undergoing follow-up cerebral angiograms (79). Blood temperature of the ipsilateral jugular venous bulb was monitored as a surrogate for local brain temperature. Clinical testing during the procedure in awake patients, and after the procedure in all patients, found that this selective cooling process was painless and produced no serious adverse effects. Transcranial Doppler monitoring and serial blood sample analyses showed stable parameters. In three following clinical studies, this direct infusion method of SEBC was applied, during ischemia and post-reperfusion, in acute ischemic stroke LVO patients (80–82). However, brain temperature or surrogates were not measured. Due to the limited number of patients and study design, the efficacy of SEBC in LVO patients remains to be determined. Nevertheless, SEBC with brief cold fluid infusion was found to be safe in LVO patients undergoing endovascular therapy and was not associated with serious adverse events. A phase II randomized controlled trial is currently being conducted to study brief SEBC with IACI in a larger group of LVO acute ischemic stroke patients undergoing clot extraction (83). In 2018, a single-arm explorative study investigating the safety of brief SEBC-IACI-induced brain cooling in anterior circulation acute ischemic stroke patients undergoing clot extraction (and refractory to tissue plasminogen activator therapy) was completed (84). The results are pending publication.

**Table 1:** Clinical studies of selective endovascular brain cooling with cold fluid infusion.

**Year**	**Author**	**N (Controls)**	**Setting**	**IA infusion**	**Sequence**	**Cold inf volume**	**Brain temp**	**Blood flow**	**Outcome**
2010	Choi^*^	18	Angiogram	ICA	Intra-proc	300 ml	YES	TCD	Clinical
2012	Neimark^**^	Data from^*^	Computer simulation	ICA	Intra-proc	300 ml	YES	NO	Clinical
2016	Peng	11 (15)	Acute ischemic stroke	CA	Pre-Revasc	500 ml	NO	NO	Infarct volume
2016	Chen	26	Acute ischemic stroke	CA	Pre/Post-Revasc	350 ml	NO	NO	Clinical
2018	Wu	45 (68)	Acute ischemic stroke	CA	Pre/Post-Revasc	350 ml	NO	NO	Infarct volume

* *First-in-human proof of concept*.

** *Data from clinical study inputted in computer simulated heat transfer model of the human head*.

*IA, intra-arterial; Inf, infusion; Temp, temperature; ICA, internal carotid artery; TCD, transcranial Doppler; CA, cerebral artery; Intra-proc, intra-procedural; Pre-Revasc, Pre-Revascularization*.

## Discussion

The advantages of endovascular selective brain cooling for the treatment of LVO stroke are mani-fold. One, with significantly reduced time to target temperature of the organ of interest, the brain, hypothermic neuroprotection can be achieved quickly. Hereby, the physical concept of SEBC is ideally suited to achieve brain cooling quickly (10–30 times faster than traditional systemic hypothermia) and with minimal invasiveness because it directly modifies the cerebral arterial input temperature and would be administered via the routine endovascular route. Two, selective brain cooling allows to reduce the impact of cooling on the body, thus minimizing or avoiding systemic hypothermia and its adverse consequences. Three, due to the endovascular route, SEBC is ideally suited to be applied as an adjunct treatment to endovascular recanalization procedures in LVO stroke. Four, due to its selective and immediate cooling features and endovascular route, SEBC would be capable to attenuate the destructive forces of reperfusion injury, locally and directly, following endovascular clot-extraction.

In practice, and taking the direct infusion method as an example, the SEBC catheter would be placed in the ipsilateral (ischemic hemisphere) internal carotid artery through the same intra-arterial access as used for clot extraction (Figure 1D). The catheter diameter would be small enough to fit through the regular guiding sheath. Selective brain cooling would be performed immediately following thrombectomy via exchange of catheters. Parallel use of thrombectomy and SEBC catheters is a possibility. In order to achieve brain hypothermia quickly, one could even attempt to cool the ischemic brain before thrombectomy is performed (intra-ischemic), as done in the explorative clinical studies (Table 1). However, this would undoubtedly delay reperfusion therapy and pose additional risks for distal embolization and cerebral vascular injury due to manipulations of the cooling catheter as it is pushed past the clot.

The key to reducing the impact of ischemia and reperfusion injury is the suppression of their pathophysiological mechanisms. While it is clear that SEBC-induced hypothermic neuroprotection would be delayed in LVO patients when they finally undergo endovascular clot-extraction, regardless of how quickly brain cooling can be achieved, the results from animal stroke models and our understanding of the molecular and cellular evolution of stroke suggest that brain cooling is neuroprotective, even when delayed for several hours. This is a plausible assumption. If the early stage excitotoxicity cannot be prevented, brain hypothermia may still suppress the later-stage inflammatory processes, reduce edema, and prevent vessels from becoming too “leaky.” Disturbance of the structural integrity and leaky vessels are considered the basic preconditions for developing post stroke ICH.

The beneficial impact of SEBC on the potential harms of sudden reperfusion is more evident when considering SEBC is applied as an adjunct to endovascular clot-extraction. Furthermore, SEBC induced via the direct mixing method with IACI could offer additional benefit as the incoming blood would be diluted, therefore reducing the impact of inflammatory promoters and immune cells on the ischemic and reperfused brain tissue. Another important advantage of the infusion method is that the dimensions of the infusion-type cooling catheter would be small enough to fit through the guiding sheath that has already been placed to navigate the EVT catheter to remove the clot, requiring only an exchange of catheters or even allowing concomitant use of both catheter systems (Figure 1D).

There are limitations of SEBC. First, although hypothermia induction would be rapid, the duration of cooling would be limited by the routine times allowed for indwelling endovascular catheters to remain in the arterial system. Second, the direct mixing method with IACI, albeit the fastest cooling method in physical terms, would be limited by the volume that could be infused within a certain period. In contrast, the extracorporeal blood cooling concept would theoretically address the issue of hypervolemia as practically the same amount of blood is re-introduced into the cerebral circulation as it was removed from the system before exposing the blood to the external heat-exchanger. With this isovolemic cooling concept, long-term brain hypothermia would become feasible. However, this isovolemic method adds layers of invasiveness and complexity to the hyperacute workflow, such as a second arterial puncture (blood outflow), vascular reconstruction (e.g., temporary arterial occlusion), necessity of a perfusion specialist, and heparinization of blood in the external circulation. Third, potential adverse effects may occur from local exposure to cold, additional fluid volume (local hemodilution) and mechanical stress from the endovascular cooling catheter. Thus, careful monitoring of vitals, dilution, local temperature, and potential vascular injury and spasm would be necessary. Lasty, there are yet no devices on the market for SEBC. Only routine catheters have been used to explore and investigate the safety and feasibility of brief SEBC (10–15 min) in elective and acute cerebrovascular patients. While the results have been promising and larger trials are underway, it is questionable whether brief and uncontrolled IACI will deliver the answers to the ideal depth, duration, and timing of brain hypothermia the determination of which should be based on physiological parameters and cerebral metabolic demand, such as changes in brain metabolism, cerebral blood flow, and infarct evolution. This could become more critical when infusing fluids distally to a cerebral artery occlusion (intra-ischemic brain cooling) without any information about the metabolic and hemodynamic condition of the ischemic tissue bed. As such, we believe that to provide a safe and most efficient SEBC, specialized catheter systems are necessary that offer excellent heat-exchange, mixing, and embedded safety and control mechanisms.

Given the growing and widespread utilization of endovascular clot-extraction in LVO stroke and lack of additional means to counter the deleterious mechanisms of ischemia and reperfusion injury (12, 85), SEBC is an appealing concept to reap the benefits of therapeutic hypothermia while minimizing the adverse effects of systemic hypothermia. Based on our current understanding of the mechanisms of stroke, reperfusion, and therapeutic (brain) hypothermia, it is reasonable to consider an improvement in outcome and reduction in the occurrence of post-stroke ICH may occur. Currently, technologies that would enable safe and controlled SEBC in acute ischemic stroke patients are in pre-clinical and explorative clinical development (Supplement Table). However, it is foreseeable that specialized medical devices for SEBC will be available in the near future. Ultimately, clinical investigations will show whether SEBC will be a safe, practical, and effective tool in the armamentarium of stroke treatment.

## Data Availability Statement

The original contributions presented in the study are included in the article/Supplementary Material, further inquiries can be directed to the corresponding author/s.

## Author Contributions

JC and JP-S contributed conception of the article. JC wrote the first draft of the article. All authors have approved the final version of the manuscript, contributed critical review and revision of the manuscript.

## Conflict of Interest

JC and JS are co-founders of Hybernia Medical, LLC. SP and JS are consultants to Hybernia Medical, LLC. The remaining authors declare that the research was conducted in the absence of any commercial or financial relationships that could be construed as a potential conflict of interest.
