# Supplementary material for: Selective Brain Hypothermia in Acute Ischemic Stroke: Reperfusion Without Reperfusion Injury
Source: Front Neurol. 2020 Nov 13;11:594289. doi: 10.3389/fneur.2020.594289 (PMC7691595; doi:10.3389/fneur.2020.594289)
Supplement: Supplementary file 1 [file Table_1.DOCX]

Supplement Table. Characteristics of selective brain cooling concepts.

| **Physical Cooling Concept** | **Location of Heat-Exchange** | **Description** | **Advantages** | **Drawbacks** | **Development Stage/Clinical Setting** |
| --- | --- | --- | --- | --- | --- |
| Convection | Endoluminal |  |  |  |  |
|  | Trans-nasal | Injection of coolant mist into the nasal cavities | Less invasive; ambulatory application feasible | Cools only forebrain; slow brain cooling; patient discomfort; mucosal irritation; short cooling duration | Clinical product available in Europe; cardiac arrest and stroke |
| Conduction | Surface |  |  |  |  |
|  | Head and Neck | Surface cooling pads placed on scalp and neck | Less invasive; ambulatory application feasible; prolonged cooling feasible | Ineffective and slow brain cooling; patient discomfort | Clinical product available for treatment of heat-stress, pain, and rehabilitation |
|  | Intravascular |  |  |  |  |
|  | Endovascular/Intra-arterial | Closed-loop catheter with circulating cold fluid in the carotid artery | No addition of fluid volume to the circulatory system; modification of arterial input temperature; prolonged cooling feasible | Requires endovascular access; slow brain cooling; limited heat-exchange capacity due to small size of carotid artery | Pre-clinical |
|  | Extracorporeal |  |  |  |  |
|  | External Heat-Exchange | Blood is removed from the system, externally cooled, then re-introduced into the cerebral circulation | No addition of fluid volume to the circulatory system except from treatment of blood in the external circulation; fast brain cooling | Extracorporeal handling and cooling of blood is complex and resource-intensive; requires additional arterial access and vascular reconstruction | Clinical application in cardiac surgery for cerebral protection |
| Mixing/Infusion | Intravascular |  |  |  |  |
|  | Transvenous | Cold fluid is infused into the jugular vein in a retrograde fashion | Theoretically, fast brain cooling possible | Requires access to the jugular vein and vascular reconstruction; cooling duration limited due to added fluid volume; retrograde infusion | Pre-clinical |
|  | Endovascular/ Intra-arterial | Cold fluid is infused into the carotid artery in an anterograde fashion | Fastest endovascular brain cooling method; direct modification of arterial input temperature; anterograde infusion; can use same access as for endovascular thrombectomy | Requires arterial access; cooling duration limited due to added fluid volume; safety and control features necessary | Pre-clinical evidence for safety and efficacy in acute ischemic stroke models; clinical investigation of safety and efficacy of cooling concept over brief duration in acute ischemic stroke patients ongoing |
